# Object detection algorithm based on improved YOLOv8 for drill pipe on coal mines

**DOI:** 10.1038/s41598-025-89019-8

**Published:** 2025-02-18

**Authors:** Xiaojun Li, Miao Li, Mingyang Zhao

**Affiliations:** 1 School of Energy Science and Engineering, Henan Polytechnic University, Jiaozuo, 454003 China; 2 Henan International Joint Laboratory of Coalmine Ground Control, Jiaozuo, 454003 China

**Keywords:** Gas extraction, Drill pipe detection, YOLOv8n, Deformable convolution, Dynamic head, Coal, Computational science

## Abstract

Gas extraction is an important measure for coal mine gas disaster control. Its effect is closely correlated to the drilling depth. The existing methods usually determine the drilling depth by manually counting the number of drill pipes, and the number of drill pipes can be automatically counted by object detection and real-time tracking algorithms. An improved object detection model was proposed for the problem of the poor performance of the object detection algorithm due to such interference factors as bright light, low illuminance and heavy dust and mist in coal mines. In terms of data augmentation, the ACE dehazing algorithm is introduced to improve image quality. In order to solve the problem of leak detection caused by the irregular shape that appears due to the interference of bright light, the deformable convolution DCNv2 module was integrated in the C2f module to make the sampling points of the convolution kernel diffuse irregularly, so as to fully extract the shape features of the drill pipe and then improve the detection rate of the model. For the problem of too low confidence of the model in detecting drill pipes due to uneven illumination, the attention paid by the model to the features of the drill pipe could be improved by embedding the SimAM non-parametric attention mechanism module in the backbone network, which can further improve the confidence of the drill pipe. For the problem of low average category detection accuracy caused by the changeable environment of the underground drilling site, the dynamic head was used to improve the ability of the model to extract the features of the drill pipe in scale, space, and channel, and improve the average category detection accuracy of the drill pipe. To address the issue of diverse angle differences between predicted and real boxes, CIoU loss function is replaced with the SIoU loss function. Finally, the improved detection algorithm was verified with the homemade drill pipe dataset. The experimental results showed that: the improved model effectively alleviated the problem of partial leak detection of the original network for scenes such as heavy dust and mist and uneven illumination; the recall rate increased by 4.9%; the mean average precision was improved by 5.3%. At the same time, it maintains a high real-time performance (the FPS is 117), providing the basis of the drill pipe detection model for the application of real-time tracking of the number of drill pipes.

## Introduction

Coal seam gas extraction is an important link in the process of coal mining, and the drilling depth plays a vital role in improving the efficiency of gas extraction and reducing the safety hazard^1^. At present, the drilling depth mainly relies on manual counting of the number of drill pipe or casing, but the underground environment is complex. There will be strong light interference, too much coal dust, uneven light and light source position movement frequently and other problems when the drilling machine is working on different drilling sites. Moreover, the manual counting has some problems such as large error, low efficiency and difficult management. Therefore, it is necessary to use the artificial intelligence object detection algorithm to realize automatic counting by means of real-time detection and tracking counting, in which the tracking effect depends on the detection accuracy of the detection algorithm^2^, But the working environment of the drilling machine leads to the problem of missing detection when the current mainstream target detection and measurement method identifies drill pipe in some scenarios. To solve this problem, it is important to create a high precision drill pipe detection model.

The traditional object detection method is not effective in the complex and changeable underground environment. However, a series of object detection models based on deep learning have been successfully applied in many aspects of coal mines, among which YOLO^3^ (You Only Look Once) takes into account both real-time and accuracy, and is widely used in coal mines. For example, Hao Shuai et al.^4^ detected foreign bodies on the conveyor belt by adding a YOLOv5 network model with depth-separable convolution and CBAM^5^ attention mechanism, which successfully improved the detection rate and accuracy of various foreign bodies. Song Liye et al.^6^ re-placed ordinary convolutional blocks with lightweight modules and used the YOLOv7^7^ network of multi-scale feature extraction pyramid network to detect mining shovel in real time, greatly reducing the number of parameters and improving the detection speed. Shao Xiaoqiang^8^ replaced the backbone network of YOLOv5s with the lightweight network, and added the self-attention mechanism module to the lightweight backbone network to improve the multi-scale feature extraction capability of the model and improve the aver-age detection accuracy of the model. Zhang Hui et al.^9^ improved the YOLOv5s backbone network and replaced the loss function to improve the convergence speed of the model. Moreover, the improved model showed good real-time detection ability and robustness in the detection of underground workers in complex environments and occlusion scenes. Zhang Mingzhen^10^ added a module containing residual blocks to YOLOv3^11^, using residual blocks to avoid the problem of gradient disappearing or gradient explosion in the training process, and effectively reduced the missed rate of pedestrian detection. Zhang Huizhao^12^ et al. demonstrated dynamic cracks in the failure process for complex cracks, combined YOLOv5 with DIC cloud image, quickly and accurately intelligently identified dynamic cracks and counted the number. Shan Pengfei et al.^13^ modified the loss function and feature extraction network based on YOLOv5, and used histogram equalization and Laplace operator to enhance the image, effectively improving the detection accuracy of thermal images of coal gangue. Liu Mingrui et al.^14^ carried out convolutional re-placement of the original YOLOv7 network to detect small targets in UAV remote sensing images, combined with the channel attention mechanism, and enhanced the feature ex-traction ability of the model for small targets. Mao Qinghua et al.^15^ improved YOLOv5s algorithm and combined a completely new method for determining the overlap between the hydraulic support guard plate and the anchor frame of the shearer drum, accelerating detection speed while improving detection accuracy. Akhil et al.^16^ integrated a pyramid of dilated convolutional layers into the feature extraction network of the YOLOv7 model and enhanced the recognition capability by adding additional YOLO detection heads, achieving accurate localization and detection of facial landmarks. Hanae et al.^17^ proposed a license plate detection and recognition method based on YOLO v8, which was combined with image processing technology and text recognition OCR technology to significantly improve accuracy, speed, and adaptability to various real-world scenarios. Ajantha et al.^18^ proposed CBS-YOLOv8 for object surface defect detection by enhancing the YOLOv8 model, introducing coordinate attention, BiFPN, and SimSPPF to improve real-time feature extraction capability and reduce computational requirements and model complexity.

To sum up, YOLO model is more widely used in coal mine scenarios and has a good effect. Moreover, the original YOLOv8n model cannot accurately detect drill pipe in some drilling roadways with complex environments, and it is difficult to meet the requirements of real-time detection and tracking. Therefore, this paper improves on the basis of YOLOv8n and proposes an improved YOLOv8n model for drilling pipe inspection in complex environments. The experimental results show that the improved model can improve the recall rate and mean average precision under the scene of uneven illumination and large dust and mist, and can identify and locate the drill pipe position more accurately. The main contributions of this study are presented as follows:

1. The primary contribution lies in the innovation of application scenarios, which applies the advanced object detection algorithm YOLO to the research of underground drill pipe detection in coal mines, filling the gap in this field and having profound significance for improving the safety, accuracy, and efficiency of coal mine production.
2. The ACE dehazing algorithm^19^ is introduced to preprocess the dataset, improve image quality, and reduce the impact of complex environmental factors in coal mines on algorithm performance.
3. In the YOLOv8n backbone network, deformable convolution DCNv2^20^ is integrated into C2f module to improve the model’s ability to extract irregular distribution features.
4. The SimAM attention mechanism^21^ is introduced to cope with the model’s attention degree to drill pipe features under uneven illumination.
5. The default detection Head of YOLOv8n is replaced with Dynamic Head^22^ to improve the model’s global extraction ability of drill pipe features to address complex scene changes.
6. In order to solve the problem of directional mismatch between the real box and the predicted box and improve the accuracy and robustness of localization, the SIoU loss function^23^ is used to replace the default CIoU loss function of YOLOv8n.

The rest of this paper is structured as: The second section mainly describes the dataset and research methods. ACE dehazing algorithm is proposed to improve image quality and optimize the dataset. The improvements made to the baseline model YOLOv8n to enhance performance are described in detail subsequently. The third section provides a detailed introduction to the entire experimental process and a specific analysis based on experimental results; The section four is regarding the discussion on this research, including the current limitations of the model and possible directions for next improvement. Finally, the conclusion of the study is given in the last section.

## Materials and methods

### Dataset

In this paper, the dataset is used as the underground drill pipe dataset of coal mines. The dataset comes from the surveillance video of the gas drilling site of a coal mine bottom pumping lane. A total of 19 drilling sites and 201 drilling holes are collected, covering various possible scenarios and situations. Due to the angle problem of some surveillance videos, the drill pipe cannot be photographed, and some surveillance videos have repeated scenes. Therefore, it is necessary to remove irrelevant or duplicate images among them. After preprocessing the dataset, set an appropriate frame extraction frequency based on the complexity of the video content and the motion speed of the drill pipe. Use the professional label tool *labelme* to label drill pipe in the extracted image, including the category (such as drill pipe) and position (such as rectangular box). Ultimately, a high-quality and standardized dataset for underground drilling pipe object detection in coal mines is obtained.

#### ACE dehazing algorithm

**Fig. 1:**
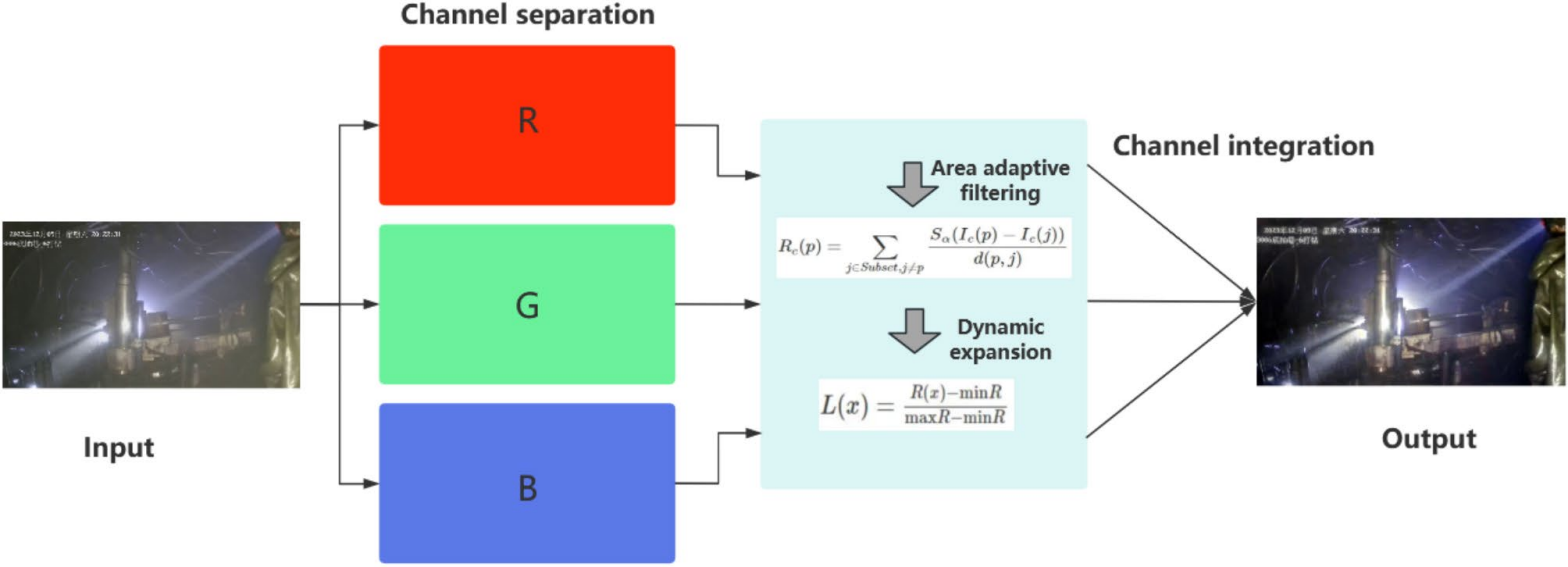
ACE dehazing algorithm flowchart.

**Fig. 2:**
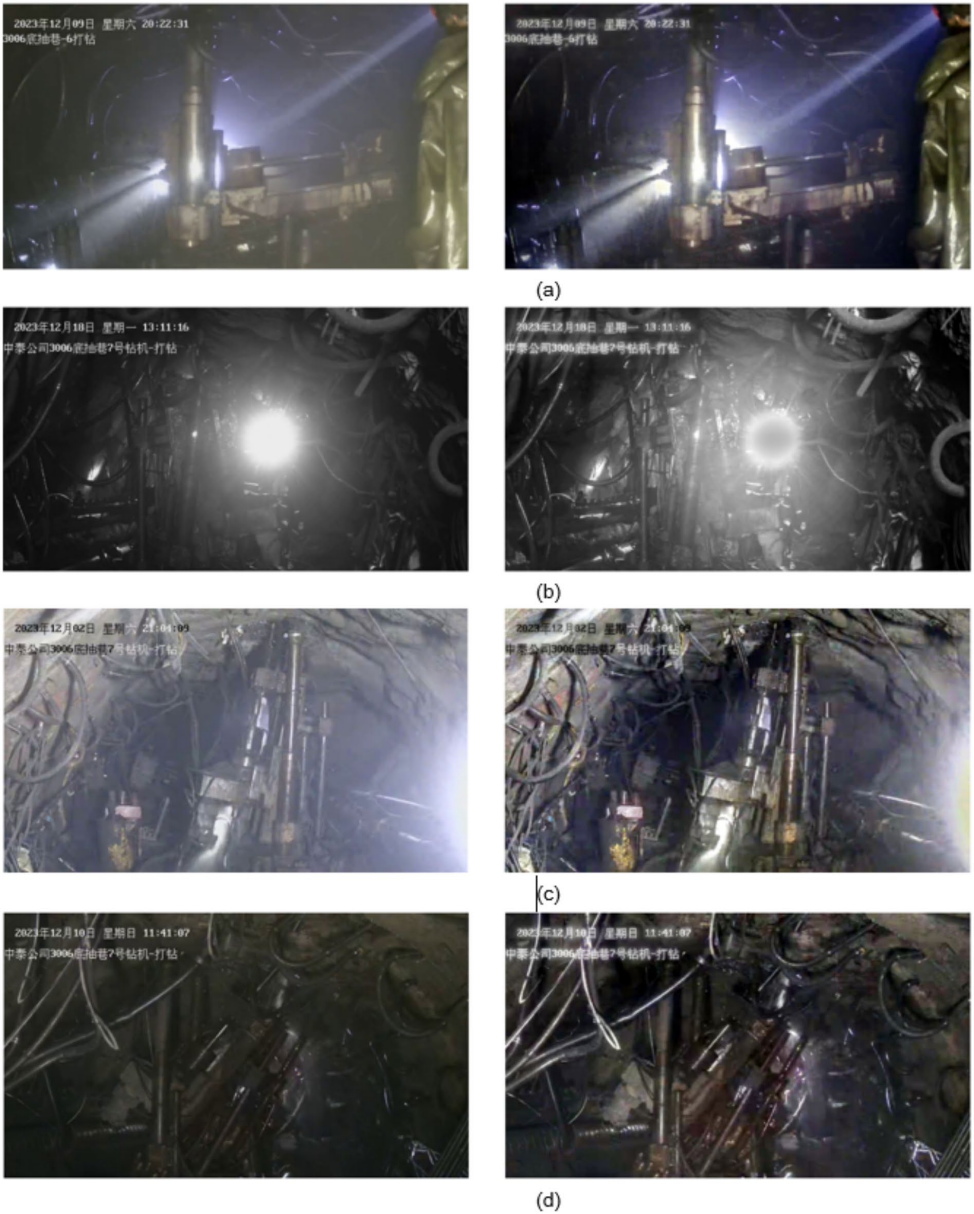
ACE dehazing algorithm performance demonstration.

For blurry images, ACE dehazing algorithm is introduced into the improved algorithm for image enhancement^19^. The quality issues of underground video surveillance in coal mines mainly include: (1) Insufficient lighting underground results in low brightness in images captured by video surveillance, which is highly detrimental to intelligent target recognition. (2) Underground lighting is primarily based on point light sources, and the complex lighting conditions lead to uneven illumination in the images captured by video surveillance, which interferes with intelligent target recognition to some extent. (3) Underground dust and mist can cause large areas of gray-white in the images, resulting in blurry target areas and affecting the recognition accuracy of the algorithm. There are three main sources of dust and mist: First, the friction between the drill rod and the coal and rock layers generates a large amount of dust and small debris, forming dust and mist. Second, drilling requires continuous flushing with water flow, and when the ventilation in some tunnels is strong, water droplets will diffuse into the air, forming water mist. Thirdly, in some scenes, unreasonable lighting intensity or light source position can cause a dust and mist effect in the image (although the actual working position does not have dust and mist). Therefore, this paper uses the ACE dehazing algorithm for image preprocessing. The ACE dehazing algorithm first separates the *R*, *G*, and *B* channels of the image, performs region-adaptive filtering calculation on the pixel point *p* in each channel, then dynamically expands the obtained calculation results, and finally obtains the enhanced image results. The process is shown in the Fig. 1:

Select some drilling field images for comparative experiments, and the comparison results are shown in the Fig. 2. For scenes with large dust and mist (a) and (c), the ACE algorithm can clearly eliminate the dust and mist effect in the image, improving the image clarity. For the point light source scene (b), the ACE algorithm can improve the visibility of dark areas while reducing the light intensity, alleviating the impact of uneven lighting. For the insufficient brightness scene (d), the ACE algorithm enhances the overall brightness of the image, revealing more image details.

### Method

#### YOLOv8n model

YOLO, as an end-to-end single-stage object detection algorithm, has become a representative algorithm for real-time detection with its fast speed since its release. YOLOv8 is a relatively high-performing model in the YOLO series, offering five different scales: n (nano-version), s (small-version), m (medium-version), l (large-version), and x (extra-large-version). Considering the requirement of real-time inspection application in mine, this paper decides to use YOLOv8n, which has the smallest volume and the fastest detection speed.

YOLOv8n mainly has three parts: Backbone network, Neck network and detection Head. The Backbone consists of Conv, Context to Fusion (C2f) and Spatial Pyramid Pooling-Fast (SPPF). Conv structure carries out convolution operations on feature maps to extract image features and perform down sampling operations. By introducing context information, the C2f structure fuses low-level features with high-level features to better capture the semantic information and spatial relationship of the object. The SPPF structure maximizes the pool of the feature map and improves the receptive field of the network. At the same time, it integrates features from the same feature map at different scales to enrich the semantic features of the feature map. The Neck part adopts the Path Aggregation Network structure^24^, which combines features of different scale feature maps by forming a bidirectional path from bottom up and top down to improve the model’s receptive field and feature fusion ability. Decoupled Head^25^ was used for the Head part to separate the feature map with two branches of regression and classification, and then fused.

#### Improved YOLOv8n drill pipe detection algorithm

**Fig. 3:**
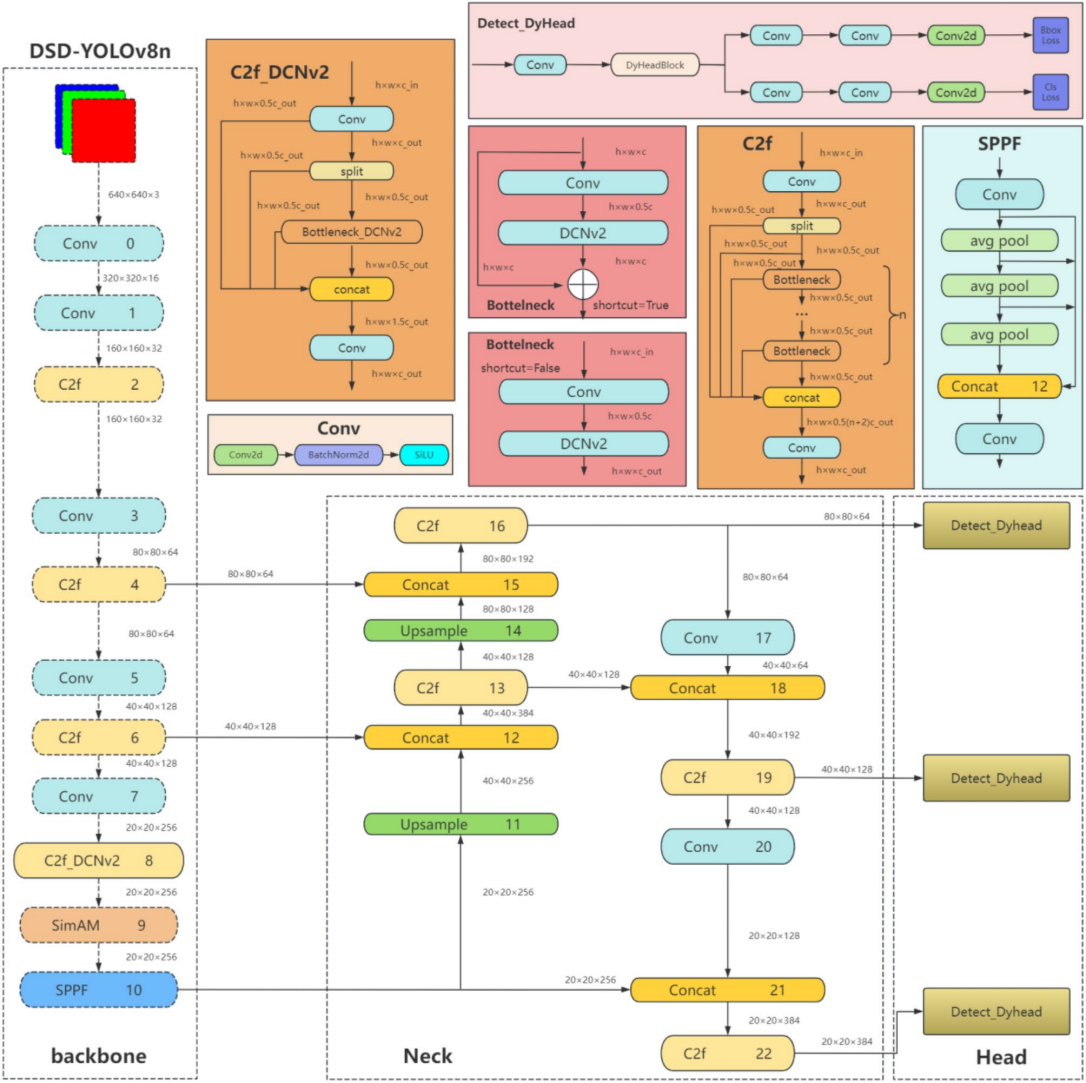
Detection framework of the proposed algorithm.

The algorithm framework proposed in this paper is shown in Fig. 3. The main improvements include: First, deformable convolution DCNv2 is used to replace the C2f module of layer 8 in the backbone network; Second, SimAM attention mechanism is embedded in the front layer of SPPF layer. Third, the dynamic head replaces the default detection head of YOLOv8n. The default CIoU loss function of YOLOv8n is replaced with SIoU loss function in the last.

##### Deformable convolution DCNv2

**Fig. 4:**
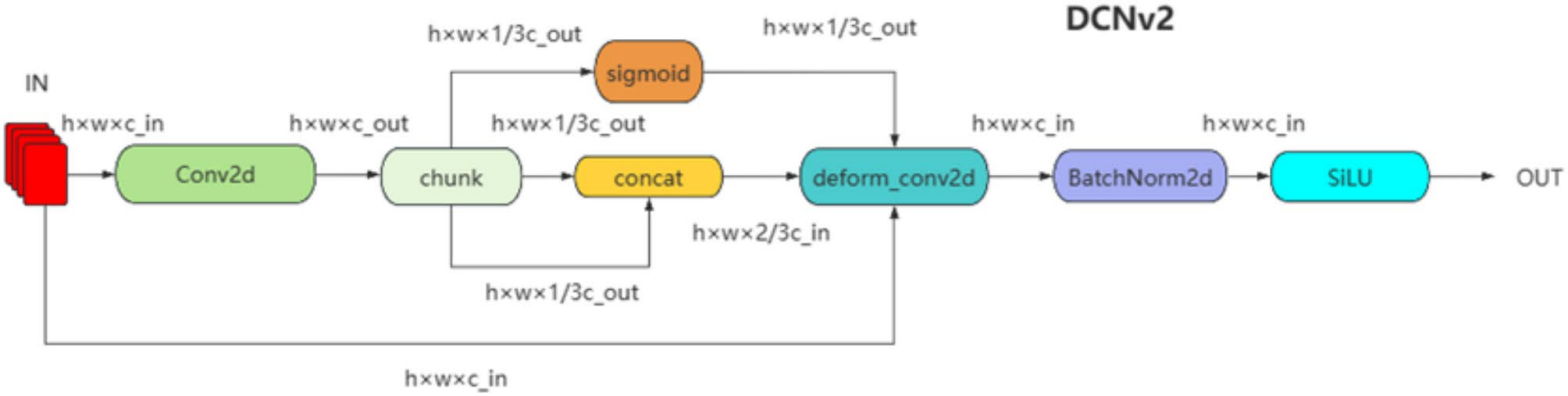
Specific structure of DCNv2.

The underground environment of the coal mine where the drill pipe is located is complex, and the drilling site images are often disturbed by strong light. Conventional convolution operations can only adjust the size of regular shapes, making it difficult for the network to extract drill pipe features effectively. To address this issue, deformable convolution (DCNv2)^20^ is introduced in the YOLOv8n model to replace the second Conv module of Bottleneck in C2f, and its network structure is shown in the upper left corner of Fig. 3. The specific structure of DCNv2 is shown in Fig. 4.

**Fig. 5:**
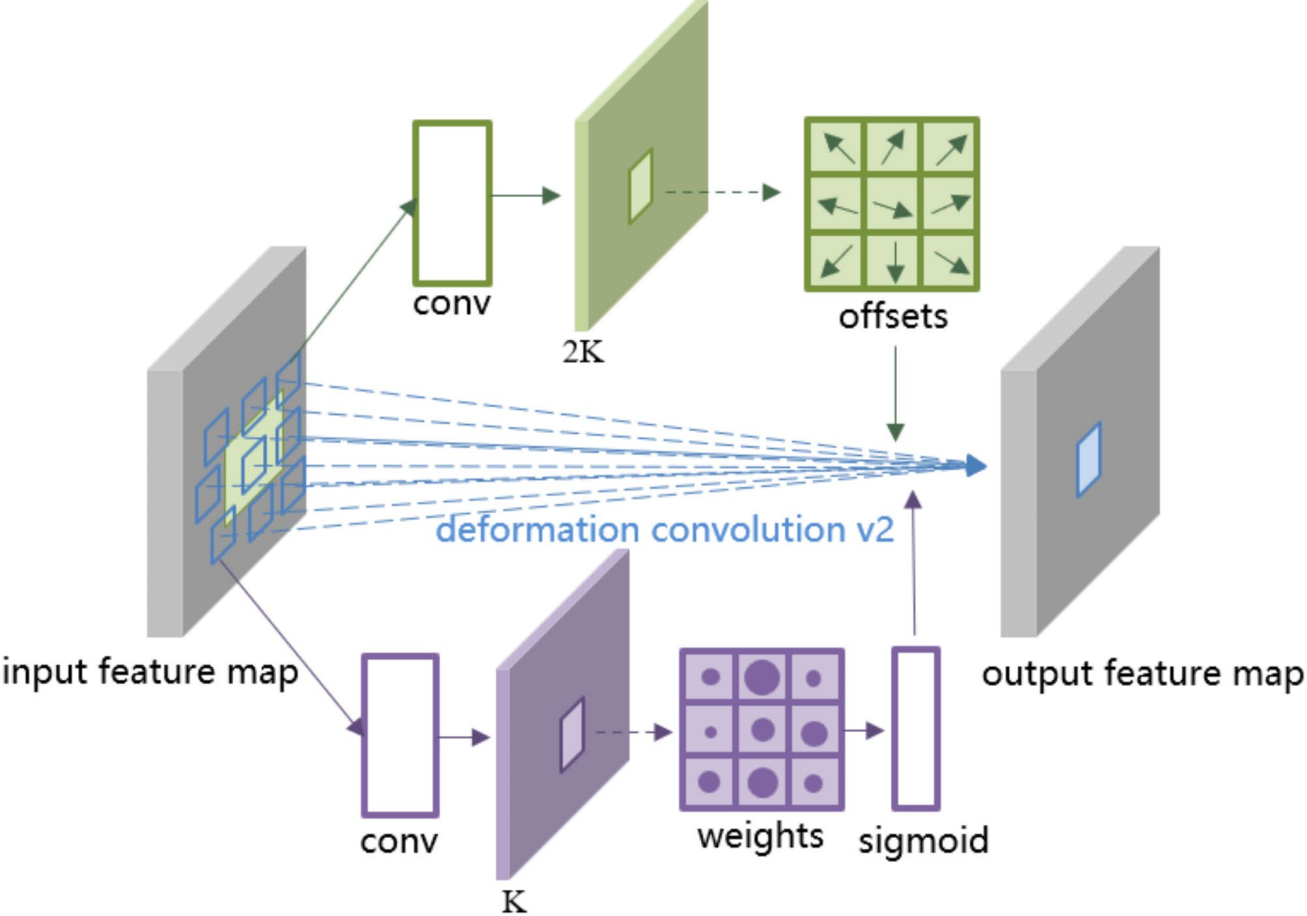
Calculation process of DCNv2.

In traditional convolution operations, the receptive field of each convolution kernel is fixed, meaning that each convolution operation samples pixels from the input feature map in a fixed manner. However, in most applications, the size, shape, and position of objects may change at any time, so traditional convolution methods cannot adapt well to these changes. Deformable Convolution can dynamically adjust the sampling position of the convolution kernel to adapt to the geometric changes of objects. It adds a learnable offset on the basis of ordinary convolution, allowing the sampling points of the convolution kernel to spread out in a non-grid shape. However, deformable convolution may extend far beyond the region of interest in terms of spatial support, which can lead to the features being influenced by irrelevant image content. Therefore, DCNv2 learns a change amplitude through modulation, limiting the spatial support range and increasing the sampling density within the region of interest. The calculation formula and process of DCNv2 are shown in formula (1) and Fig. 5, respectively.

1 $$\begin{array}{*{20}{c}} {y\left( {{p_0}} \right)=\mathop \sum \limits_{{{p_n} \in R}} W\left( {{p_n}} \right) \cdot X\left( {{p_0}+{p_n}+\Delta {p_n}} \right) \cdot \Delta {m_n}} \end{array}$$

Where $\Delta {p_n}$ and $\Delta {m_n}$ represent the learnable offset and modulation scalar of the nth position, respectively. $\Delta {m_n} \in \left[ {0,1} \right]$, the initial value of $\Delta {m_n}$ is 0.5, $\Delta {p_n}$ is a real number with no range constraint, and its initial value is 0.

Firstly, a convolution operation is performed on the input feature map. The upper branch produces an offset field with *2 K* channels, corresponding to the offsets in the *X* and *Y* directions, while the lower branch generates a modulation field with *K* channels to obtain the weight coefficient feature map. This feature map is then fed into the sigmoid layer to obtain the modulation values. Finally, the output is a feature map with *3 K* channels.

##### SimAM attention mechanism

**Fig. 6:**
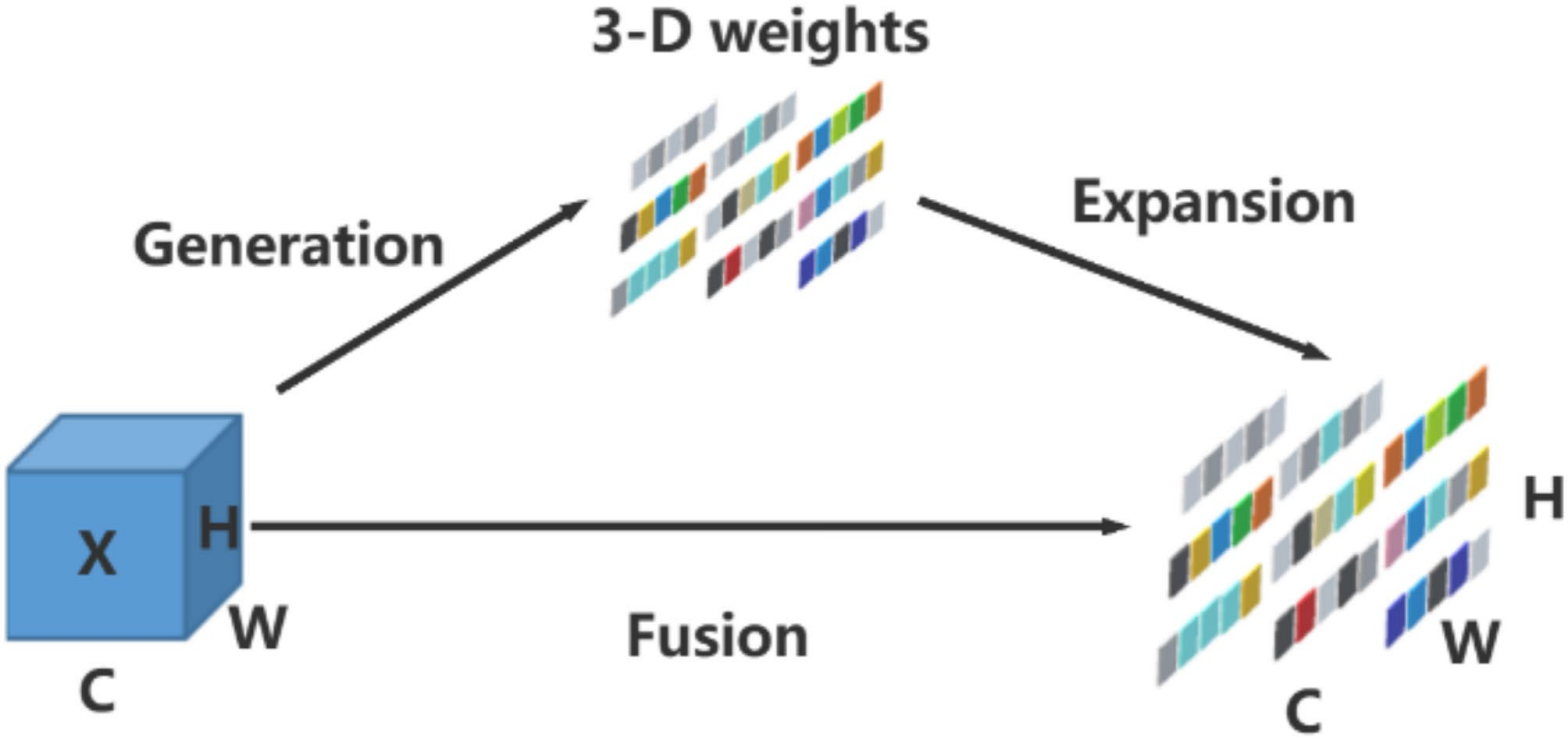
Schematic diagram of SimAM.

In order to solve the problem of uneven illumination caused by moving light source in coal mine, SimAM attention mechanism^21^ is introduced. The SimAM attention machine is a 3D weighted attention mechanism without introducing additional parameters, which reduces the computational overhead. The SimAM attention mechanism combines channels and spaces^26^ to assign unique weights to each neuron, as shown in Fig. 6.

The SimAM attention mechanism proposes that the importance of neurons in the feature map is different, that information-rich neurons usually exhibit different firing patterns than peripheral neurons, and that activated neurons usually inhibit peripheral neurons, known as spatial inhibition. Neurons with spatial inhibitory effects should be given higher importance. Measuring the linear separability between neurons is one way to determine the activation of neurons, and its calculation formula is shown in formula (2).

2 $$\begin{array}{*{20}{c}} {{e_t}\left( {{\omega _t},{b_t},y,{x_i}} \right)={{\left( {{y_t} - \hat {t}} \right)}^2}+\frac{1}{{M - 1}}\mathop \sum \limits_{{i=1}}^{{M - 1}} {{\left( {{y_0} - {{\hat {x}}_i}} \right)}^2}} \end{array}$$

Where t and ${x_i}$ are the target neurons and other neurons in the single channel of the input feature map, where $\hat {t}={\omega _t}t+{b_t}$and ${\hat {x}_i}={\omega _t}{x_i}+{b_t}$ is the line transformation of t and ${x_i}$, and $M=H \times W$ is the number of neurons in the feature map channel. ${\omega _t}$ and ${b_t}$ are transformations of the weights and biases of the feature graph channels. To simplify the equation, we take values 1 and − 1 for ${y_t}$ and ${y_0}$, respectively, as shown in formula (3).

3 $$\begin{gathered} {e_t}\left( {{\omega _t},{b_t},y,{x_i}} \right)=\frac{1}{{M - 1}}\sum\limits_{{i=1}}^{{M - 1}} {{{\left( { - 1 - \left( {{\omega _t}{x_i}+{b_t}} \right)} \right)}^2}} \\ +{\left( {1 - \left( {{\omega _t}t+{b_t}} \right)} \right)^2}+\lambda \omega _{t}^{2} \\ \end{gathered}$$

Where ${\omega _t}$ and ${b_t}$ can be obtained by formulas (4) and (5).

4 $$\begin{array}{*{20}{c}} {{\omega _t}= - \frac{{2\left( {t - {\mu _t}} \right)}}{{{{\left( {t - {\mu _t}} \right)}^2}+2\sigma _{t}^{2}+2\lambda }}} \end{array}$$

5 $$\begin{array}{*{20}{c}} {{b_t}= - \frac{1}{2}\left( {t+{\mu _t}} \right){\omega _t}} \end{array}$$

Where ${\mu _t}$ and $\sigma _{t}^{2}$ are the mean and variance of all other neurons of this channel except neuron *t*, respectively. It is reasonable to assume that all pixels of a channel follow the same distribution. Based on this assumption, the mean and variance are avoided from double calculation, which is very important to reduce the computational overhead. Then the minimum energy formula is:

6 $$\begin{array}{*{20}{c}} {e_{t}^{*}=\frac{{4\left( {{{\hat {\sigma }}^2}+\lambda } \right)}}{{{{\left( {t - \hat {\mu }} \right)}^2}+2{{\hat {\sigma }}^2}+2\lambda }}} \end{array}$$

Where $\hat {\mu }$ and ${\hat {\sigma }^2}$ are the mean and variance of all neurons of this channel. The lower energy neuron is more distinct from the surrounding neurons, and the more visually important it is. Therefore, the importance of each neuron can be obtained by $\frac{1}{{e_{t}^{*}}}$. We use scaling factors to represent the effect of attentional mechanisms on neuronal responses. Then the effect formula is shown in formula (7).

7 $$\begin{array}{*{20}{c}} {\tilde {X}=sigmoid\left( {\frac{1}{E}} \right) \odot X} \end{array}$$

Where *E* groups $e_{t}^{*}$ in channel and spatial dimensions, and the *sigmoid* function is used to constrain values that are too large in *E.*

The operations in the SimAM attention mechanism are element-by-element and can be done in just a few lines of code, making it easy to embed SimAM in the YOLOv8 model. In this paper, the SimAM attentional mechanism is added in front of the SPPF module, so that the network can better pay attention to the feature neurons of the drill pipe during the detection process, so as to reduce the impact of light changes.

##### Dynamic head

An excellent object detection head needs to achieve three aspects: scale-awareness, spatial-awareness, and task-awareness. In order to have better expression, a unified framework is designed, namely the Dynamic Head (DyHead)^27–29^. In response to the complexity and variability of the underground environment in coal mines, the default detection head in the YOLOv8 structure is replaced with the Dynamic Head. This modification processes the feature map from three aspects: scale, spatial, and task, reducing the impact of background changes on drill pipe features.

**Fig. 7:**
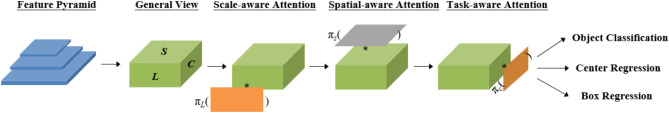
Schematic diagram of DyHead.

DyHead adds a Conv layer and DyHeadBlock module to the YOLOv8 default detection head. The structure of Detect_DyHead module is shown in the upper right corner of Fig. 3, and the basic schematic diagram of DyHead is shown in Fig. 7.

*L* different-scale features are extracted from the pyramid backbone network. Scale adjustments are made using up-sampling and down-sampling, and *S* is defined as *H×W*. The features are represented as *FεR*^*L×S×C*^. DyHead combines the three dimensions using a multi attention mechanism to construct a single detection head and maximize its performance. For a given feature *F*, the generalized form of self-attention is as shown in formula (8).

8 $$\begin{array}{*{20}{c}} {W\left( F \right)=\pi \left( F \right) \cdot F} \end{array}$$

Where $\pi \left( \cdot \right)$ is an attention function. A simple way to handle this attention function is through the fully connected layer. However, directly learning the attention function across all dimensions incurs a high computational cost. Therefore, DyHead transforms the attention function into three sequential attention mechanisms, with each attention mechanism responsible for one dimension, as shown in formula (9).

9 $$\begin{array}{*{20}{c}} {W\left( F \right)={\pi _C}\left( {{\pi _S}\left( {{\pi _L}\left( F \right) \cdot F} \right)} \right) \cdot F} \end{array}$$

**Fig. 8:**
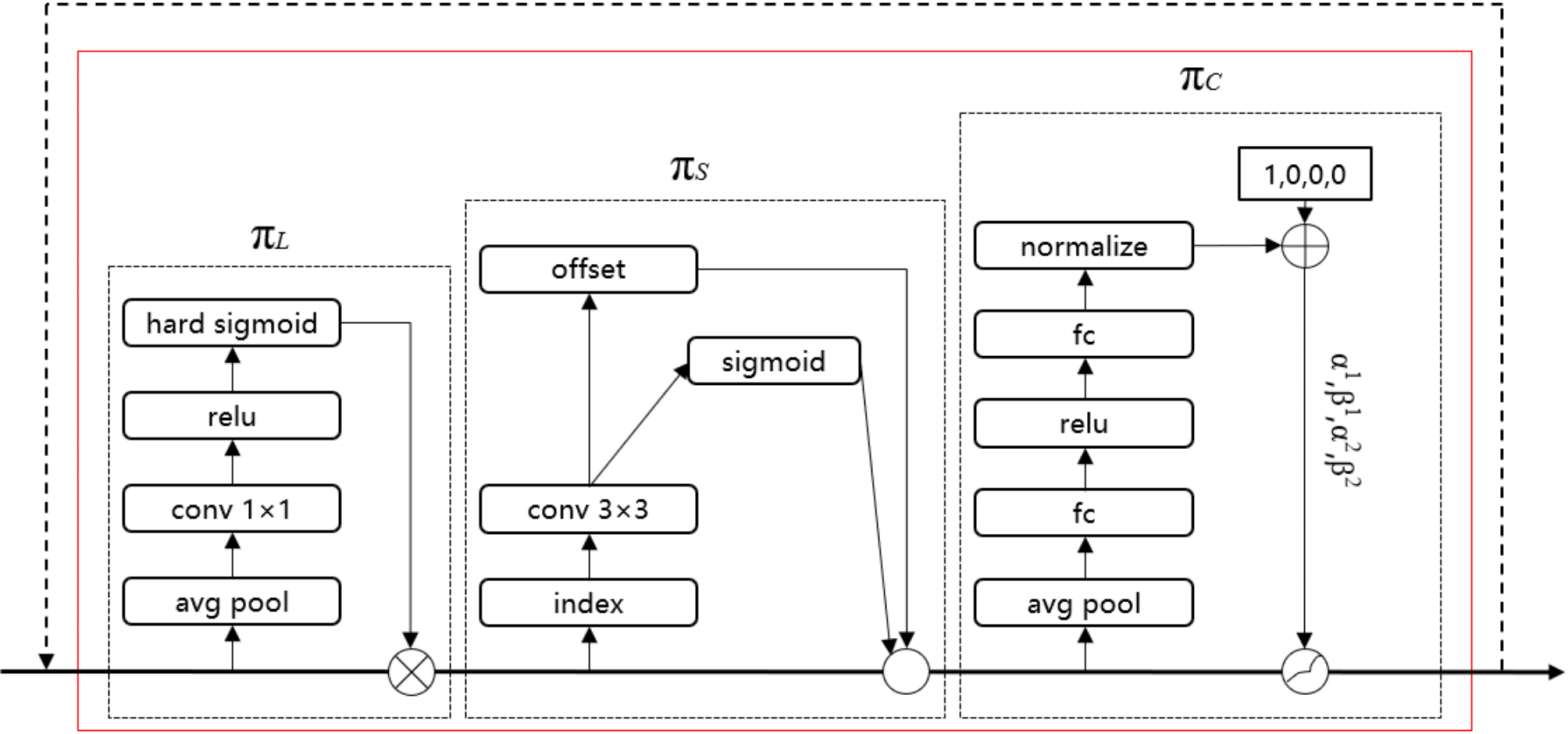
Detailed design of DyHead.

First, a scale-aware attention mechanism ${\pi _L}\left( \cdot \right)~$is introduced to dynamically fuse features from different scales based on their semantic importance. Then, a spatial location-aware attention module ${\pi _S}\left( \cdot \right)~$is introduced to focus on the discriminative ability at different spatial locations. To achieve joint learning and improve generalization, a task-aware attention mechanism ${\pi _C}\left( \cdot \right)~$is deployed at the end. After completing the attention decomposition, DyHead further implements cascading by stacking the three attention mechanisms in the above order. The detailed configuration of DyHead is shown in Fig. 8.

##### SIoU loss function

**Fig. 9:**
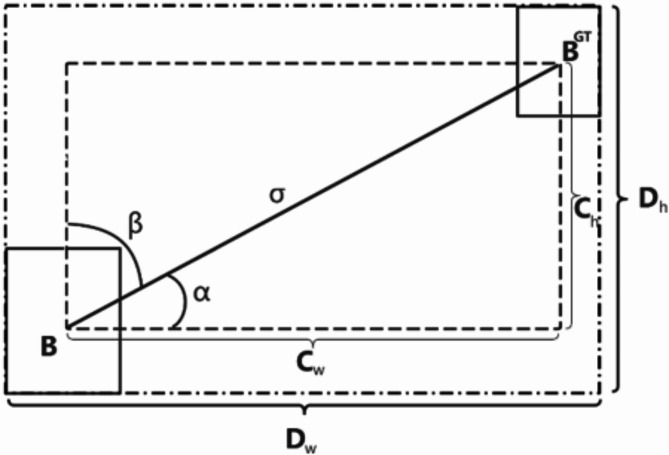
Angular cost.

The default loss function used by YOLOv8 is CIoU, however, the model trained using CIoU does not meet the preset performance indices in terms of recall rate and mean average precision. Therefore, CIoU is replaced with SIoU. The SIoU loss function further considers the problem of directional mismatch between the real box and the predicted box based on CIoU, and redefines and adjusts the SIoU loss function accordingly^23^. The function consists of four components, among which the angle loss specifically describes the minimum angle formed between the center point’s connecting line and the x-y axis, as shown in Fig. 9.

When the center point is aligned on the x-axis or y-axis, ∧= 0. When the center point is connected to the x-axis at 45 °, ∧= 1. This punishment mechanism can effectively guide the predicted box to move towards the nearest position on the coordinate axis of the object box, while reducing the degrees of freedom of distance related variables in the prediction process, thereby improving the accuracy of the prediction. Given the predicted box and the real bounding box, the angle between the center line of the two boxes and the horizontal axis is considered as α, and the angle with the vertical axis is considered as β, which is the vertical distance between the centers of the two bounding boxes. If α < π/4, the model attempts to minimize α, otherwise minimize β, where β = π/2 – α.

10 $$\begin{array}{*{20}c} {\Lambda = 1 - 2 \times \sin^{2} \left( {\arcsin \left( x \right) - \frac{\pi }{4}} \right)} \\ \end{array}$$

where $$x=\frac{{{c_h}}}{\sigma }=\sin (\alpha )$$.

11 $$\begin{array}{*{20}{c}} {\sigma =\sqrt {{{(b_{{cx}}^{{gt}} - {b_{cx}})}^2}+{{(b_{{cy}}^{{gt}} - {b_{cy}})}^2}} } \end{array}$$

12 $${c_h}=\hbox{max} (b_{{cy}}^{{gt}},b_{{cy}}^{{gt}}) - \hbox{min} (b_{{cy}}^{{gt}},b_{{cy}}^{{gt}})$$

13 $${c_w}=\hbox{max} (b_{{{c_x}}}^{{gt}},{b_{{c_x}}}) - \hbox{min} (b_{{{c_x}}}^{{gt}},{b_{{c_x}}})$$

Distance cost describes the distance between center points, and its penalty cost is positively correlated with the angle cost. When α → 0, the contribution of distance cost is greatly reduced. On the contrary, the closer α is to π/4, the greater the contribution of distance loss. The formula is as follows:

14 $$\begin{array}{*{20}{c}} {\Delta =\mathop \sum \limits_{{t=x,y}} \left( {1 - {e^{ - \gamma {\rho _t}}}} \right)} \end{array}$$

$$where~{\rho _x}={(\frac{{b_{{{c_x}}}^{{gt}} - {b_{{c_x}}}}}{{{D_w}}})^2},~{\rho _y}={(\frac{{b_{{{c_y}}}^{{gt}} - {b_{cy}}}}{{{D_h}}})^2},~\gamma =2 - \Lambda .$$

Shape cost is defined by calculating the difference in width between two boxes and the maximum width ratio between them (the same applies to length processing):

15 $$\begin{array}{*{20}{c}} {\Omega =\mathop \sum \limits_{{t=w,h}} {{(1 - {e^{ - {\omega _t}}})}^\theta }} \end{array}$$

$$where~{\omega _w}=\frac{{\left| {w - {w^{gt}}} \right|}}{{\hbox{max} (w,{w^{gt}})}}~,~{\omega _h}=\frac{{\left| {h - {h^{gt}}} \right|}}{{\hbox{max} (h,{h^{gt}})}}~.$$

IoU cost is the maximum intersection over union between the predicted box and the real box, as shown in the following formula:

16 $$\begin{array}{*{20}{c}} {IoU=\frac{{\left| {B \cap {B^{GT}}} \right|}}{{\left| {B \cup {B^{GT}}} \right|}}} \end{array}$$

The final defined box regression function is:

17 $$\begin{array}{*{20}{c}} {{L_{box}}=1 - IoU+\frac{{\Delta +\Omega }}{2}} \end{array}$$

## Experiment and result analysis

### Environment configuration

The hardware and software platform configurations in the experiment are shown in Table 1.

**Table 1:** Hardware and software platform configurations.

Configuration name	Version parameter
Operating system	Ubuntu 22.04.5 LTS
Programming language	Python 3.8
GPU	NVIDIA GeForce RTX 4090 24G
CPU	Intel^®^ Xeon^®^ Platinum 8352 V
Deep learning framework	Pytorch2.0.0
Cuda version	11.8

### Evaluation metrics

This paper uses Precision, Recall, mean Average Precision (mAP50), and Frames Per Second (FPS) as performance evaluation metrics. Among them, the mAP50 is the evaluation metric with the highest importance weight for this model, followed by Recall. The FPS is the main evaluation metric for model speed to judge real-time detection performance. When FPS > = 30, the model satisfies the requirements for real-time detection.

The precision *P* is the ratio of correctly predicted results among all positive samples, as shown in formula (18).

18 $$\begin{array}{*{20}{c}} {P=\frac{{TP}}{{TP+FP}}} \end{array}$$

The recall *R* is the correctly predicted ratio of all positive samples, as shown in formula (19).

19 $$\begin{array}{*{20}{c}} {R=\frac{{TP}}{{TP+FN}}} \end{array}$$

The mean Average Precision *mAP*50 is the average *AP* of each category under the condition whose *IoU* threshold is 0.5, and *AP* is the area surrounded by *P-R* curve and coordinate axis, as shown in formulas (20) and (21).

20 $$\begin{array}{*{20}{c}} {AP=\mathop \smallint \limits_{0}^{1} P\left( r \right)dR} \end{array}$$

21 $$\begin{array}{*{20}{c}} {mAP50=\frac{1}{n}\mathop \sum \limits_{n}^{i} AP\left( i \right)} \end{array}$$

Where *r* is the value of recall, $P\left( r \right)$ is the precision value corresponding to recall, and *n* is the number of categories.

Frames Per Second (FPS) takes the total number of inferable images in one second and its unit is frame.

### Model training

**Fig. 10:**
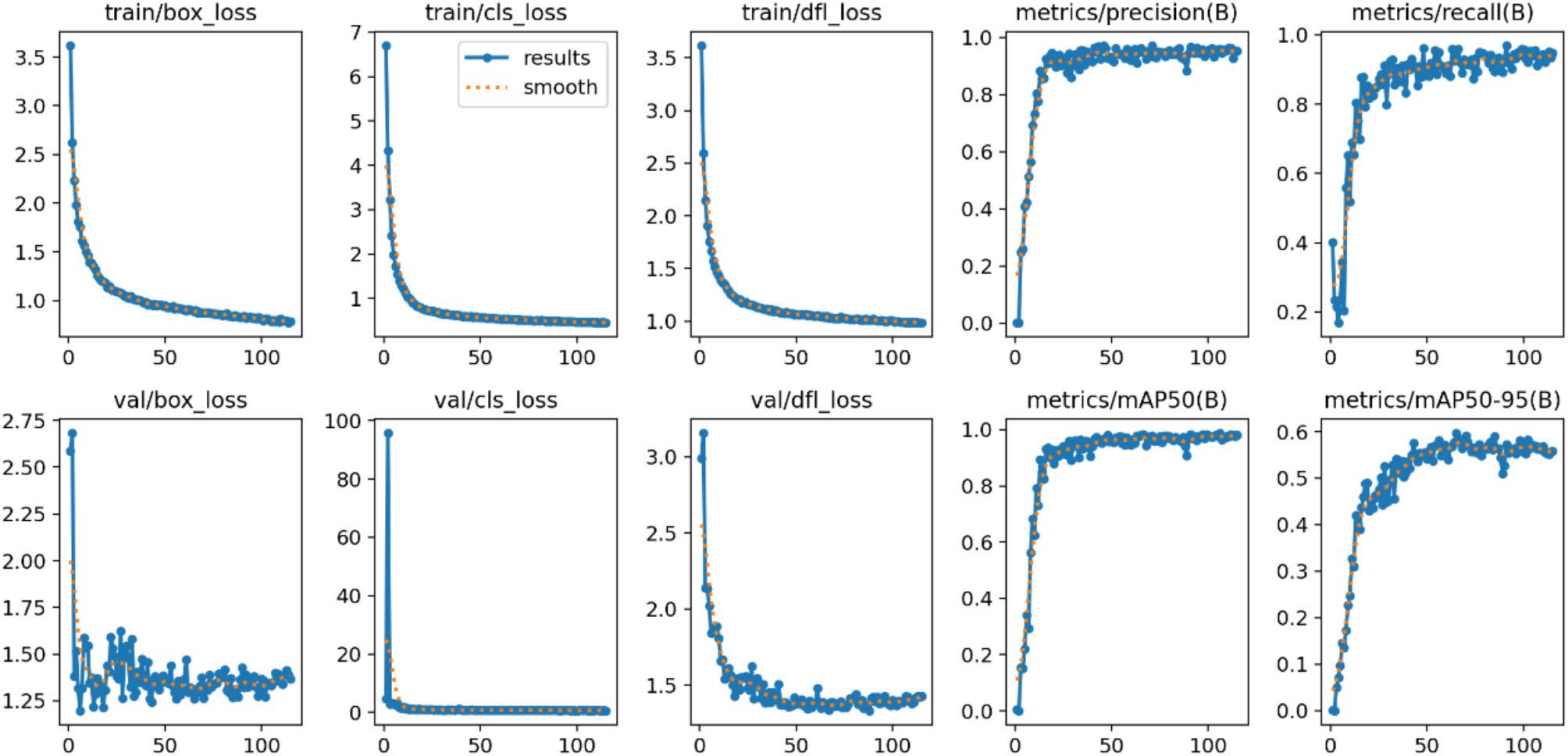
Loss and metrics representation of the improved algorithm.

**Fig. 11:**
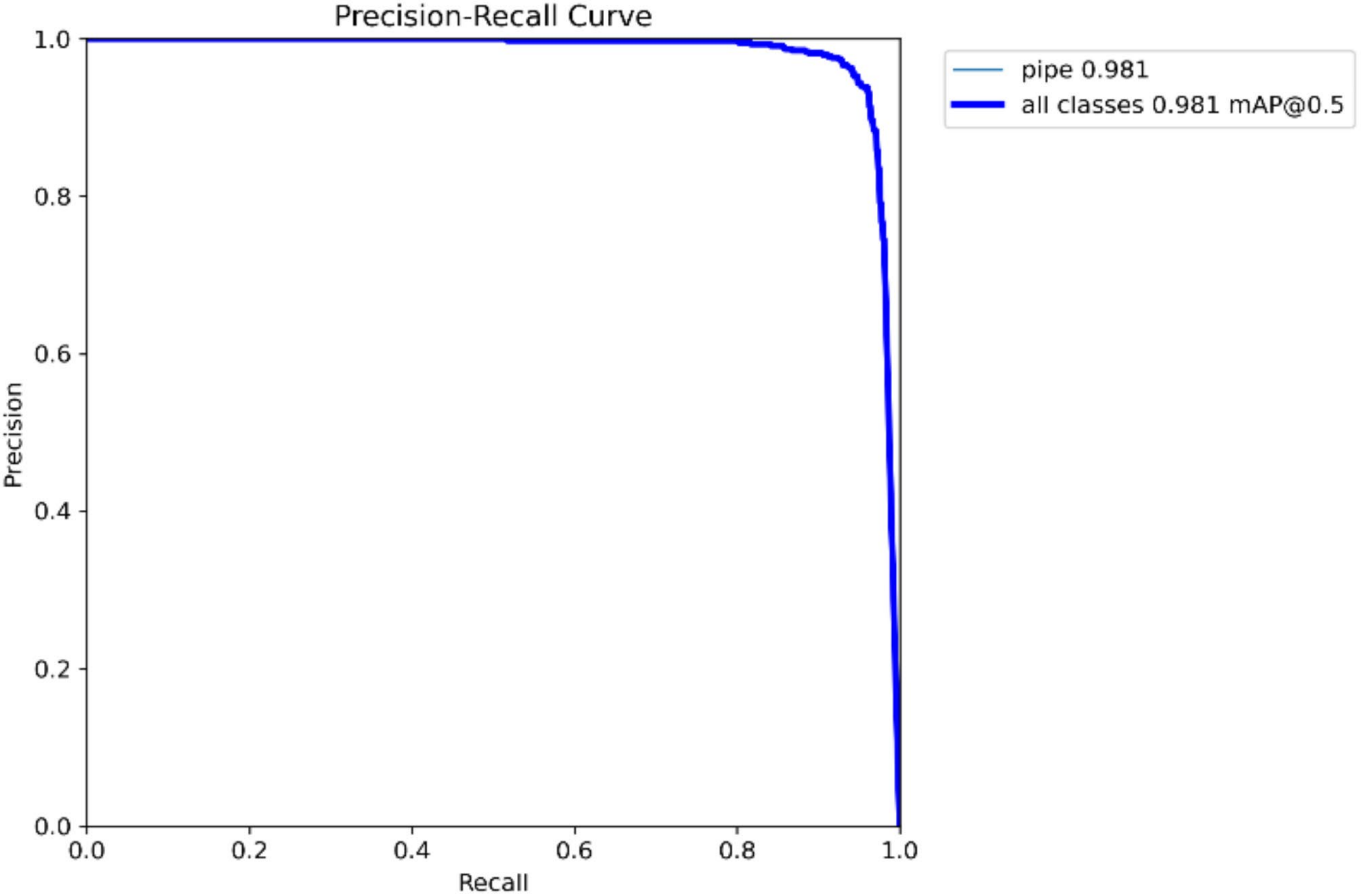
P-R curve (mAP50 value) of the improved algorithm.

Regarding the training parameters of the model, the input image size is 640 × 640, training rounds are 200, batch size is 16, initial learning rate is set to 0.01, and final learning rate is set to 0.01. The optimizer is Adam, the momentum factor is set to 0.937, and the attenuation coefficient is 0.005. Model training is based on YOLOv8n. The loss value and metrics changes of the improved algorithm is shown in Fig. 10. The horizontal axis represents the training epochs. As the epoch increase, the loss gradually decreases and various metrics gradually improve. Within the 0–20 epoch range, both show rapid changes, especially in the category loss function graph of the validation set. Since it is a single-class detection, the rate of decrease is particularly significant. Between epochs 20 and 50, the speed of change begins to slow down, and each loss function starts to converge towards their minimum values, while the metrics also tend to converge towards the highest value. After 50 epochs, enter a slow and repetitive adjustment state. Figure 11 represents the obtained result of the mAP50 for the improved algorithm, as it shows the mAP50 value achieved 0.981. The improved algorithm has indeed demonstrated better performance than the baseline model YOLOv8n.

### Ablation experiment

In order to verify the effect of each improvement, an ablation experiment was conducted, and the results are shown in Table 2.

**Table 2:** Ablation experiment table.

Model	ACE	DCNv2	SimAM	DyHead	SIoU	*P*	*R*	mAP50
1	×	×	×	×	×	0.978	0.867	0.928
2	√	×	×	×	×	0.985	0.869	0.940
3	√	√	×	×	×	0.982	0.881	0.947
4	√	√	√	×	×	0.991	0.893	0.953
5	√	√	√	√	×	0.979	0.895	0.966
6	√	√	√	√	√	0.985	0.916	0.981

Model 1 in the table is the detection result of the original YOLOv8n network. Models 2, 3, 4, 5 and 6 are improved models after sequentially adding ACE dehazing algorithm, DCNv2, SimAM, DyHead and SIOU. It is obvious that the baseline model YOLOv8n, without any improved components, is the worst performance indices. Every improvement is necessary for the model as they have demonstrated a positive effect on the final detection results. Compared with the original model, the final model achieves a 0.7% increase in precision, a 4.9% increase in recall and a 5.3% increase in mAP50. Although the precision reaches the highest value of 0.991 when the SimAM attention mechanism is added to the upper layer of SPPF layer, but mAP50 and recall have higher weights than precision. Moreover, the integration of all modules significantly improves the performance of the original YOLOv8n model.

### Comparison experiment

To further verify the advantages of the algorithm in this paper, it was compared with nine algorithms, Faster R-CNN^30^, SSD^31^, YOLOv3^32^, YOLOv5s, YOLOv7^33^, YOLOv9t^34^, YOLOv10n, YOLO11n, and the baseline model YOLOv8n.

Faster R-CNN is an improvement over R-CNN and Fast R-CNN, which enhances speed and accuracy by using a new technology called Region Proposal Network (RPN). However, it is not efficient enough for real-time applications because of substantial computational resources, and has poor detection performance for small objects. SSD was proposed by Wei et al. in 2016, with the core idea of using a single neural network architecture to simultaneously classify and locate objects in input images, and detect targets at different scales. The YOLO series algorithm has now become a representative of real-time object detection. YOLO was first proposed by Joseph et al. in 2016. Since then, it has undergone extensive research and development by various teams, developing into the 11th version, YOLO11. Due to its excellent speed performance, it has gradually become a representative of real-time object detection. This paper selects several typical YOLO algorithms, including YOLOv3, YOLOv5s, YOLOv7, YOLOv8n, YOLOv9t, YOLOv10n, and YOLO11n, and compares them with the improved algorithm. The comparison results are shown in Table 3.

**Table 3:** Comparison results of various algorithms.

Algorithm	*P*	*R*	mAP50	FPS
Faster R-CNN	0.964	0.880	0.914	19
SSD	0.952	0.874	0.910	42
YOLOv3	**0.992**	0.899	0.930	33
YOLOv5s	0.975	0.901	0.927	267
YOLOv7	0.966	0.892	0.920	61
YOLOv9t	0.981	0.895	0.933	109
YOLOv10n	0.958	0.843	0.931	168
YOLO11n	0.983	0.899	0.948	141
YOLOv8n	0.978	0.867	0.928	**464**
This paper’s algorithm	0.985	**0.916**	**0.981**	117

Significant values are in bold.

By comparing and analyzing the index data in Table 3, YOLOv3 achieves an accuracy of 0.992, which is the highest value among the compared algorithms and slightly higher than the 0.985 of the improved algorithm in this paper. The Recall is 0.916, which is higher than other compared algorithms. Also, the Recall is the evaluation index with the highest importance weight among the compared algorithms. In addition, the mAP50 of the improved algorithm in this paper reached a maximum of 0.981, demonstrating excellent performance. In terms of FPS, it is 117. Among all comparison algorithms, it is only at an intermediate level, but it is still greater than 30 frames per second, which can still fully meet the needs of real-time detection.

**Fig. 12:**
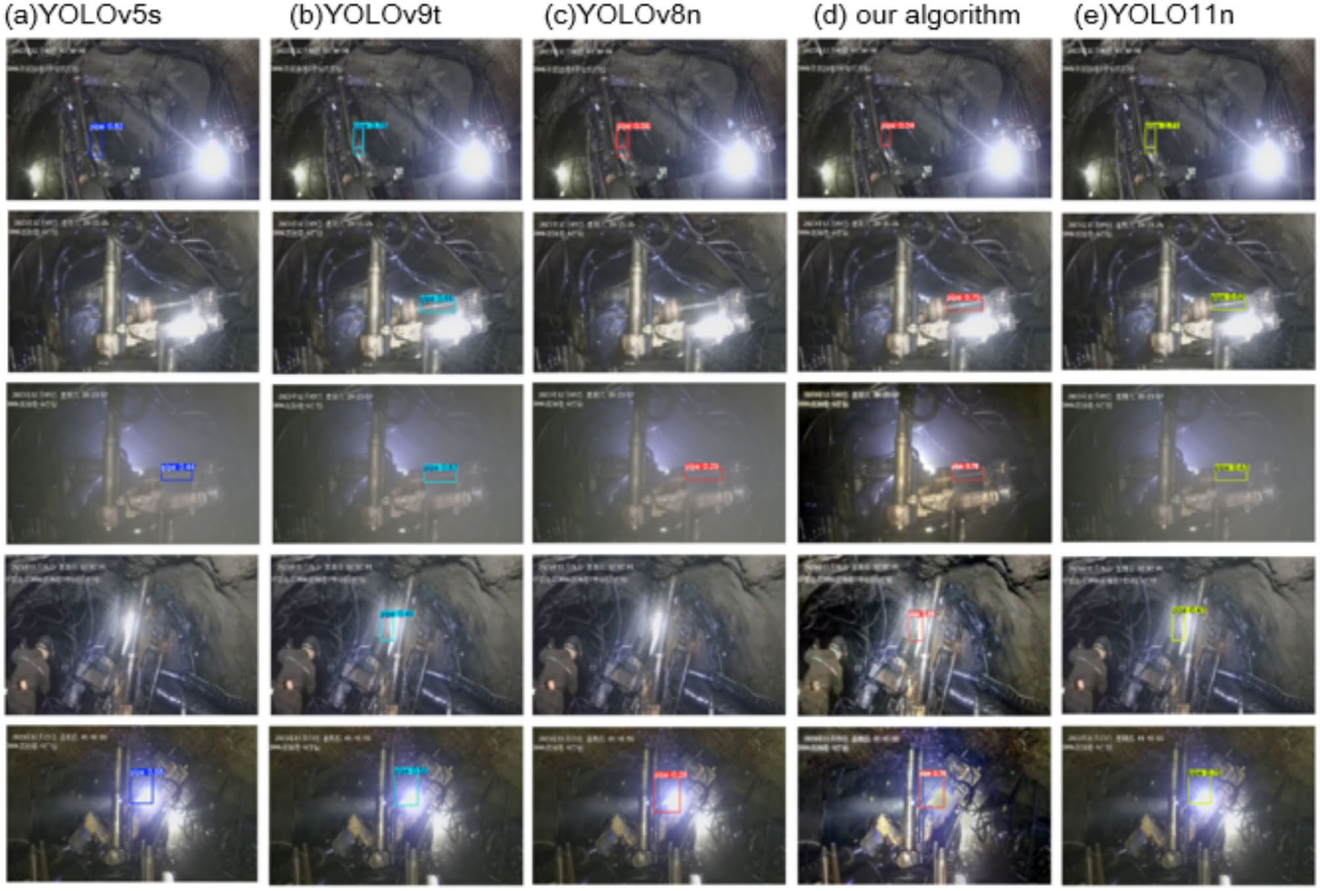
Visual comparison results.

Figure 12 shows the visual comparison results of the improved algorithm with YOLOv5s, YOLOv9t, YOLOv8n, and YOLO11n in several classic drill pipe scenarios. In the first scenario, multiple light sources and strong light directly hitting the drill pipe caused interference. YOLOv5s performed relatively well, but YOLOv9t, YOLOv8n, and YOLO11n exhibited false detection issues, with the same drill pipe being detected twice and the impact drill head being misidentified as part of the drill pipe. The improved algorithm introduces the dynamic detection head to enhance model’s ability to extract drill pipe features in terms of scale and space, improves the recognition level for non-drill pipe features, and effectively prevents false detection. In the second and fourth scenarios, strong light directly hitting the surface of the drilling machine caused strong light reflection, resulting in uneven illumination on the drill pipe surface, with missed detections in YOLOv5s and YOLOv8n, and low confidence in YOLOv9t and YOLO11n. The algorithm proposed in this paper incorporates the SimAM attention mechanism, allowing the model to better focus on high-weight feature points and enhancing its ability to perceive drill pipe features under different lighting conditions. Excessive light caused part of the drill pipe to become invisible and take on an irregular shape in the fourth scenario. In this paper, the improved algorithm uses DCNv2 with diffusible sampling points to extract feature samples of the drill pipe in an irregular manner around the center of the sampling position, effectively enhancing the model’s ability to detect features of irregularly shaped drill pipe. In the third scenario, there is a lot of dust and mist, resulting in dim lighting and unclear drill pipe features. As a result, multiple algorithms show poor detection confidence. To address this, the algorithm proposed in this paper introduces the ACE dehazing algorithm, which enhances image clarity and improves the detection performance. These algorithms perform not bad in the fifth scenario, and our algorithm is second only to YOLO11n. Overall, the detection performance of the algorithm in this paper still shows significant advantages.

## Discussion

Drill pipe, as one of the key components in coal mining machinery equipment, its detection is of grSeat significance for ensuring the safe operation of mine equipment and improving production efficiency. The development of drill pipe object detection in underground coal mines based on computer vision and deep learning is an important research direction in coal mine safety production and intelligent management. This paper introduces the deformable convolution DCNv2, SimAM attention mechanism, dynamic head, and SIoU loss function based on the YOLOv8n model, and optimizes the dataset using the ACE dehazing algorithm. The final improved algorithm shows a significant increase in both mean average precision and recall, proving that the addition of these modules is effective. However, the current algorithm is definitely not the optimal solution. There is still greater room for progress worth further discussion.

- Lightweight design. As shown in the comparison results, although the improved algorithm still meets the real-time standard, the FPS (frames per second) has significantly decreased compared to the original model and other models, which is an important point that cannot be ignored. Based on this, we consider the next step of further exploring the direction of convolutional modules and network structures, and thinking about whether we can adopt more lightweight modules or backbone networks to reduce the number of parameters and computational cost. For example, MobileNet and EfficientNet, where MobileNet is a lightweight backbone network achieved through the use of depthwise separable convolution^35^ modules.
- TensorRT acceleration. In terms of improving real-time speed, we are also considering whether we can perform TensorRT quantization on the current model for acceleration. TensorRT is an efficient library provided by NVIDIA, specifically designed to accelerate deep learning inference. It significantly improves inference speed through techniques such as mixed-precision computing and hardware acceleration. It supports a variety of popular deep learning frameworks and is widely used in real-time inference, edge computing, image recognition and other applications.
- Dataset expansion. The quality and scale of the dataset directly impact the training performance and generalization ability of the model. The drill pipe dataset in this study still has the problem of insufficient data, which leads to poor generalization ability of the model. Therefore, we plan to expand the dataset by two to three times its current size before proceeding with further training and analysis. At the same time, we will collect new drilling site data from different scenarios, which may help improve the model’s generalization ability. Additionally, data augmentation techniques such as translation, rotation, and splicing can further enrich the dataset.

## Conclusion

In this paper, a detection and recognition algorithm specifically for underground coal mine drill pipes is proposed. This algorithm takes YOLOv8n as the baseline model. The ACE dehazing algorithm is introduced to process and optimize the dataset in the first. In terms of network structure, the deformable convolution DCNv2 is integrated into C2f to extract the features of drill pipe with irregular shape effectively. By introducing the SimAM attention mechanism, the model can pay more attention to the drill pipe features in the scene of uneven light intensity and further improve the detection precision. Additionally, the default detection head of YOLOv8n is replaced by dynamic head, which enhances the ability of the model to extract drill pipe features under complex scene transformation. The default CIoU loss function has also been replaced by SIoU loss function, improving the accuracy of box regression in object detection.

The experimental results show that compared with the original YOLOv8n model, the improved algorithm has a recall rate increased by 4.9%, a mean average precision increased by 5.3%, and the FPS is 117, which fully meets the needs of real-time detection. The comprehensive performance is also better than that of Faster R-CNN, SSD, YOLOv3, YOLOv5s, YOLOv7, YOLOv9t, YOLOv10n and YOLO11n. The final obtained results are relatively excellent and satisfactory. In future work, in response to the decrease in FPS for the improved algorithm, the focus will be on further enhancing speed. This could involve using more lightweight modules or backbone networks, or using TensorRT for acceleration, in order to meet the fast response requirements in real-time scenarios. Moreover, how to build a larger, more diverse, and more representative dataset is also a key issue in future work. It is of great significance to promote the development of intelligent and safe production in underground coal mines.

## Data Availability

Data are contained within the article.
